# Post-entry blockade of small ruminant lentiviruses by wild ruminants

**DOI:** 10.1186/s13567-015-0288-7

**Published:** 2016-01-06

**Authors:** Leticia Sanjosé, Helena Crespo, Laure Blatti-Cardinaux, Idoia Glaria, Carlos Martínez-Carrasco, Eduardo Berriatua, Beatriz Amorena, Damián De Andrés, Giuseppe Bertoni, Ramses Reina

**Affiliations:** Instituto de Agrobiotecnología (CSIC-Universidad Pública de Navarra-Gobierno de Navarra), Avda, Pamplona, 123, 31192 Mutilva-Navarra, Spain; Animal Health Department, Regional Campus of International Excellence “Campus Mare Nostrum”, Universidad de Murcia, 30100 Murcia, Spain; Institute of Virology and Immunology, Bern, Switzerland

## Abstract

Small ruminant lentivirus (SRLV) infection causes losses in the small ruminant industry due to reduced animal production and increased replacement rates. Infection of wild ruminants in close contact with infected domestic animals has been proposed to play a role in SRLV epidemiology, but studies are limited and mostly involve hybrids between wild and domestic animals. In this study, SRLV seropositive red deer, roe deer and mouflon were detected through modified ELISA tests, but virus was not successfully amplified using a set of different PCRs. Apparent restriction of SRLV infection in cervids was not related to the presence of neutralizing antibodies. In vitro cultured skin fibroblastic cells from red deer and fallow deer were permissive to the SRLV entry and integration, but produced low quantities of virus. SRLV got rapidly adapted in vitro to blood-derived macrophages and skin fibroblastic cells from red deer but not from fallow deer. Thus, although direct detection of virus was not successfully achieved in vivo, these findings show the potential susceptibility of wild ruminants to SRLV infection in the case of red deer and, on the other hand, an in vivo SRLV restriction in fallow deer. Altogether these results may highlight the importance of surveilling and controlling SRLV infection in domestic as well as in wild ruminants sharing pasture areas, and may provide new natural tools to control SRLV spread in sheep and goats.

## Introduction

In the last century we have witnessed the emergence of acquired immunodeficiency syndrome, multidrug-resistant tuberculosis and tick-borne related diseases as a result of the interactions between humans and zoonotic pathogens in a pathway including wildlife and domestic animals [1].

Small ruminant lentiviruses (SRLV) infection is present in sheep and goats from Europe [2], America [3–5], Australia [6], Africa [7] and Asia [8, 9]. Economic impact of SRLV infection, highly dependent on environmental factors, breed/individual susceptibility, production system, farming practices and age of culling is often underestimated and still under study [10]. The premature removal of infected animals and the consequent increased replacement rate is a major consequence of SRLV infection. SRLV infected sheep have shown decreased fertility and number of lambs per birth, as well as a reduction of birth weight and weight gain from birth to weaning [11, 12]. Animals with advanced disease present a significantly reduced body weight at slaughter, and their carcass may not qualify for human consumption [13]. The most obvious positive result observed following the eradication of SRLV infections in goats’ herds is the disappearance of clinical cases of carpal arthritis and the improved health of the flocks [10]. This combined with the elimination of a viral infection showing a negative impact on milk production [14–16] may well explain the financial success of a combined eradication campaign comprising SRLV, such as “The Norwegian Healthier Goats program” [17].

SRLV are able to cross inter-species barrier thereby infecting sheep and goats through horizontal and lactogenic routes [10]. Since the first descriptions of natural transmissions of Visna Maedi virus (VMV) to goats, or Caprine arthritis encephalitis virus (CAEV) to sheep [18], many research groups have reported cross-species transmission in different countries [19–21]. Recently, new genotypes, subtypes and recombinant SRLV viruses have been described widening significantly their genetic and antigenic heterogeneity, likely conferring them a wider spectrum of cell and host tropism. *Env* and LTR genomic regions have been related to cell tropism by modifying the receptor usage [22] or by enhancing the promoter activity depending on the transcription factors present in a particular cell type, respectively [23]. Typically, the virus exists in the infected host as a continuum of related but divergent genetic variants called quasispecies that compartmentalize in different tissues or body fluids [24, 25] potentially favoring cross-species transmission.

The transmission of infectious agents from reservoir animal populations, often from domesticated species to wildlife in shared pastures or breeding areas (spill-over), may lead to the emergence of a range of infectious diseases in the wildlife. Spill-over is particularly important for endangered species and may also occur from wildlife to domestic animals (spill-back) affecting animal production [1]. A well-known example of adaptation to a new host is the human immunodeficiency virus (HIV) that successfully overcame the intrinsic restriction factors constitutive of the species-specific barrier, to successfully infect humans [26]. HIV-2 is a human adapted variant of the simian immunodeficiency virus (SIV) from Sootey mangabeys (*Cercocebus atys*) [27], and HIV-1 may be similarly derived from a chimpanzee (*Pan troglodytes*) SIV [28]. There are different examples of inter-species transmission of lentiviruses displaying more attenuated/virulent phenotype in the new host. In general, SIV cause no observable disease in their natural hosts but may become severe pathogens in novel host species [29]. In the case of BIV, which is non-pathogenic for *Bos taurus*, the transmission to *Bos javanicus* resulted in lethal Jembrana disease [30].

During the last decades many species of wild ruminants have been reintroduced and others have expanded their population across Europe, both in density and geographical range. Transmission of pathogens from or to domestic ruminants poses serious problems since infected wildlife and domestic ruminants may represent a pathogen reservoir to each other [31]. So far, SRLV have been found in Alpine ibexes (*Capra ibex*) from French Alps in contact with domestic goats as well as in hybrids derived from this contact, both of which showed proviral sequences related to CAEV present in local domestic goats [32]. In Rocky Mountain goats (*Oreamnos americanus*), CAEV is able to be transmitted by lactogenic and horizontal routes causing a severe multisystemic disease involving lungs, central nervous system and joints [33]. Mouflon (*Ovis aries musimon*) and domestic sheep hybrids are also susceptible to CAEV infection [34]; however, among a 101 wild mouflon population from Spain, none showed positive serology against VMV [35]. Similar results were obtained when analyzing sera from red deer in California [36–38], suggesting lack of susceptibility of these wild ruminants to SRLV.

This study aims at providing further evidence of the potential susceptibility of wild ruminants to SRLV infection. We present serological evidence that different wild ruminants may have mounted an antibody response to SRLV. Additionally, we explored the susceptibility of red- and fallow deer cells to different SRLV strains for entry, integration, replication and production of infectious viral particles.

## Materials and methods

### Animals and samples

**Figure 1:**
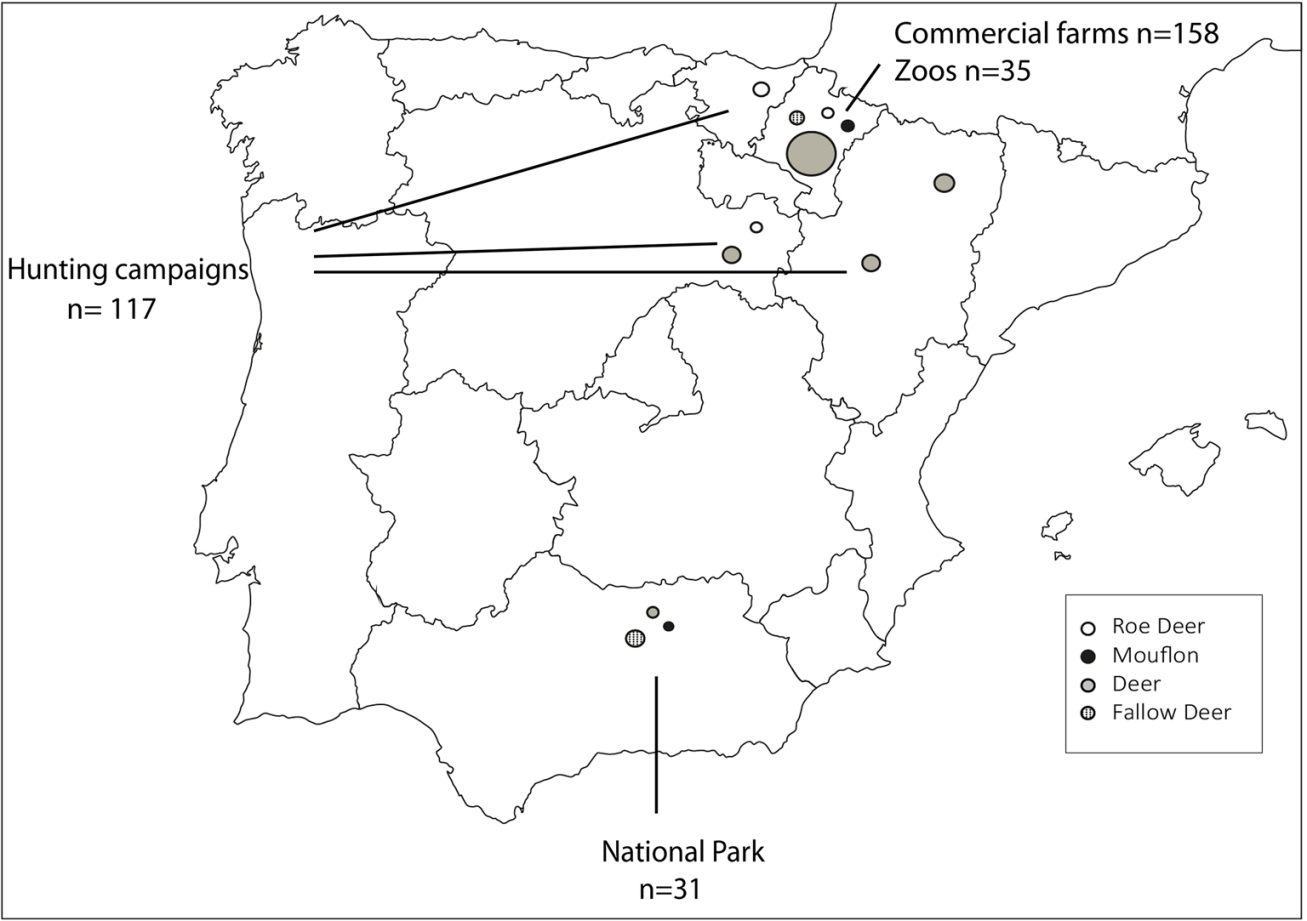
**Bubble chart of the sampling distribution.** Blood samples from red deer, fallow deer, roe deer and mouflon were obtained from commercial farms, zoological or national parks or hunting campaigns in different Spanish regions. The bubbles are sized according to the number of sampled animals.

259 red deer (*Cervus elaphus*), 36 roe deer (*Capreolus capreolus*), 40 fallow deer (*Dama dama*) and 16 mouflon (*Ovis aries musimon*) from different origins as specified in Figure 1, were sampled to obtain whole blood in EDTA-3 K as anticoagulant. Deer were from zoos (4%), national parks (4%), production farms (61%) and hunting campaigns (31%). Roe deer were all from hunts, fallow deer from zoos (37.5%) and national parks (62.5%) and mouflon were from zoos (62.5%) or national parks (37.5%).

Plasma was obtained and stored at −20 °C for serological determinations and buffy coat for PCR analyses. Peripheral blood leucocytes (PBLs) were isolated from buffy coat samples by density gradient centrifugation and resuspended in PBS for DNA extraction using QIAamp^®^ DNA Blood Mini Kit (Qiagen).

### Serological analysis

Plasma samples were analyzed for SRLV antibodies by four different ELISA methods. Commercial ELISAs for detection of SRLV antibodies were slightly modified in order to detect IgG from wild ruminant species. This was achieved in a first round, by using protein Pierce Purified Recomb^®^ Protein G Peroxidase conjugated (Recombinant Protein G, Pierce) or secondly, with a secondary antibody able to cross-react with a wide range of ruminant species, including red deer and fallow deer (EG5, Ingenasa). ELISA plates used included commercial Chekit (AG-CHEKIT CAEV/MVV kit, IDEXX Switzerland), ELITEST (Elitest-MVV Hyphen-Biomed, France) and a previously described home-made ELISA that is based on coating with single synthetic peptides alone or in combination [39]. Procedures were carried out following manufacturer’s instructions with the exception of the conjugate antibody which, as mentioned, was substituted by a secondary antibody able to react with wild species’ IgG.

### PCR

DNA samples from fifteen seropositive and four seronegative animals were subjected to amplification by described PCRs for SRLV and BIV detection (Table 1). Thermal amplification protocols were adapted to annealing temperature of each primer pair and results were analyzed in 1% agarose gels. Bands of the expected molecular weight were cut and purified using ATP Gel/PCR extraction kit (ATP Biotech Inc.). Purified products were cloned into pGEMT-easy vector (Promega) following manufacturer’s instructions and sequenced using M13 primers.

**Table 1:** Primers covering gag, pol, LTR and env regions, were used for the SRLV detection by PCR

PCR assay	Primers	Sequence 5′–3′	References
Gag nested PCR	GAG F1	TGG TGA RKC TAG MTA GAG ACA TGG	[64]
	GAG F2	CAA ACW GTR GCA ATG CAG CAT GG	
	POL R1	CAT AGG RGG HGC GGA CGG CAS CA	
	POL R2	GCG GAC GGC ASC ACA CG	
Pol nested PCR	P28	CAT GAA GAG GGG ACA AAT CAG CA	[18]
	P34	TAC ATT GGG TGC CTG GAC ATA A	
	P32	TAC CTG DGT TGG TCC YWG CCA CT	
	P33	CTT CCC AVA GTA CCT GDG TTG GTC	
	P29	GGT GCC TGG ACA TAA AGG GAT TC	
	P35	GCC ACT CTC CTG RAT GTC CTC T	
Gag-Can nested PCR	CAEV F0	AAC TGA AAC TTC GGG GAC GCC TG	[5]
	CAEV F1	AAG GTA AGT GAC TCT GCT GTA CGC	
	CAEV F2	TGG TGA GTC TAG ATA GAG ACA TGG	
	CAEV R0	GTTATCTCGTCCTAATACTTCTACTGG	
	CAEV R1	TTT TTC TCC TTC TAC TAT TCC YCC	
	CAEV R2	GGA CGG CAC CAC ACG TAK CCC	
	MVV F0	AAG TAA GGT AAG AGA GAC ACC TAC TGG	
	MVV F1	TAG ATA GAG ACA TGG CGA AGC AAG CTC	
	MVV F2	GAC AGA AGG GAA CTG TCT ATG GGC	
	MVV R0	GGT GGT GCT TCT GTT ACA ACA TAG G	
	MVV R1	GGA CGG CAC CAC ACG TGG	
	MVV R2	CCC CTC CTG YTG TTT CCC TG	
BIV nested PCR	POL3	ACA ACG GGC CGT GCT TTA CTG	[65]; [66]
	POL4	CCT CTT CCT CTA TTA CTG CTG C	
	POL5	GAR AAT CTA TGT AAG TAT CTG GG	Unpublished data (Nadin-Davis)
	POL6	CTG TTY CTT ACG TAA CAC CAC T	
	P09	CAC TGG ACG AGA TGA GGT AGT	[67]
	P06	TGG TAG TCT GAT AAA TGG CA	
	P04	CAG GCT CTT AAG GAA ATT GT	
	P11	CCA TCC TTG TGG TAG AAC ATT	
Craft-Oslo	Craft-Fw	TGA CAG AAG GAA ATT GTY TRT GG	
	Oslo-Rv	GGC ATC ATG GCT AAT ACT TCT AA	[68]
LTR PCR	LTR-Fw	TGA CAC AGC AAA TGT AAC CGC AAG	[69]
	LTR-Rv	CCA CGT TGG GCG CCA GCT GCG AGA	
CO qPCR	612-F	GAA CTC AGC CAC AAG AGG AAG AA	[70]
	613-R	CCT GCG GCA GCT ACT ATT GC	
	614 Probe	FAM AACTAGCATAATGACCAAGCCAACGCCTCT BHQ-1	
A4 qPCR	Fs7631/Fg6221	ACA AAC TGG ACC ACC ATG CA	[21]
	Rg6221	CTA GTG TTC CAT TTC CTG TTC TGT TTA	
	Probe g6221	FAM GGC AAC TGT TCW CAG TGT GTT AAT GYA ACG BHQ-1	
A4-SU PCR	SU5-1F	GTA GAT GTG TAC AAA GAC CAG	[21]
	SU5-1R	CTG CCT CTA ACA CTT GCT GC	
CO-SU PCR	B1-SU5-1R	TGC CTC TAA CAC ATC CTG CTG TGC	[70]
	B1-SU5-1F	GGT GGA ACA TAT GAC AGG AG	

Primers able to amplify bovine inmunodeficiency virus were included

### Cells and SRLV in vitro infection

Red deer (DSF) and fallow deer (FSF) skin fibroblasts were obtained from skin biopsies, isolated by trypsin disruption and cultured in DMEM medium supplemented with 10% foetal bovine serum (FBS), 1% l-glutamine and 1% antibiotic/antimycotic mix (Sigma-Aldrich). Previously obtained ovine skin fibroblasts (OSF) or goat synovial membrane cells (GSM) were used as control cells as indicated throughout the experiments.

Blood derived macrophages (BDM) from deer and fallow deer were also obtained as described [40]. Briefly, buffy coat diluted in PBS was loaded onto Ficoll-Paque Premium 1.084 (GE Healthcare) gradient to isolate peripheral blood mononuclear cells. BDM were obtained by adherence and cultured in RPMI 1640 supplemented with 1% l-glutamine, 1% non-essential amino acids, 50 µM β-mercaptoethanol, 1% vitamins, 10 mM sodium pyruvate and 10% foetal bovine serum (FBS). Sheep BDM were also obtained for comparative purposes. Cultures with fibroblast-like cells overgrowth were discarded.

Established cultures were maintained and used in experimental infections with Ev1 [41], CAEV-Co [42], A4 [21] and Ov496 strains of SRLV [19]. In vitro infection was carried out at 0.1 and 1 TCID_50_/cell in DMEM 2% medium in the case of Ev1 and 496 strains and with 20 copies/cell in the case of CAEV-Co and A4 isolates. After 16 h, cells were washed three times with PBS and resuspended in lysis buffer AL (Qiagen) for DNA extraction and amplification of viral DNA with described primers (Table 1). For confirmatory purposes, DNA from in vitro infected fibroblastic cells was subjected to PCR with primers Craft-Oslo, A4-SU and CO-SU, for strains EV1, 496 and A4, CAEV-Co, respectively (Table 1). Amplicons were purified, cloned into pGEMT-easy vector and sequenced (StabVida). Additionally, after washing with PBS, cultures were maintained 10 days post inoculation for RT-activity determinations following manufacturer’s instructions for Ev1 and 496 strains (HS-Lenti RT Activity Kit, Cavidi). Viral quantification (viral RNA copies/µL) of A4 and CAEV-Co strains in the supernatants was done by qPCR (Qiagen QuantiTec Probe RT-PCR) using primers and TaqMan probes g6621 and 614 respectively (Table 1).

BDM supernatants were collected at 3, 10 and 14 days and the CAEV-Co and A4 viral RNA was quantified by real time PCR using specific primers and Taqman probes (Table 1).

### LTR promoter activity

LTR basal activity from strains A4 [21], KV1772 [43], Ov496 [19] and CAEV-Co [42] was assayed using a luciferase reporter system. Briefly, DSF, FSF and GSM cells (10^5^ in 24-well plates) were transfected with 200 ng of each LTR construction using 4 µL of Lipofectamine-LTX Reagent and 0.2 µL of PLUS-Reagent (Life Technologies). Plasmids pGL4.13 [luc2/SV40] and “empty” p-GL4.10 [luc2] were used as positive and negative controls, respectively. At the same time, 20 ng of pGL4.73 [hRluc/SV40] Vector (Promega) were cotransfected, so that the firefly activity was standardized according to the Renilla luciferase activity. After 24 h, cells were harvested with Passive Lysis 5X Buffer (Promega), and firefly and renilla luciferase activity were measured following manufacturer’s instructions. Results were expressed as: Relative Luminiscence Units (RLU) firefly luminescence/RLU Renilla luminescence.

### Entry assay

CAEV (including human alkaline phosphatase, AP) [22] virions pseudotyped with ENV proteins from strains Ev1 [41], CAEV-Co [42], 697 [44], Roccaverano [45] and Seui [46] of SRLV, as well as Vesicular Stomatitis virus protein G (VSV-G) produced in 293-T cells as described [22, 47] were used to transduce red deer and fallow deer skin fibroblasts. Briefly, pseudoviruses were incubated with target cells and after 72 h cells were washed three times with PBS and endogenous AP activity blocked by heating at 65 °C 1 h in a humidified chamber. After blocking, NCTBI reagent was added to cells for 2–24 h and finally reaction was stopped with normal water. Stained cells were visualized and counted under light microscopy by two experts.

### Western blot

Sodium dodecyl sulfate–polyacrylamide gel electrophoresis (SDS-PAGE) was carried out using Ev1 and 496 infected DSF, FSF and OSF extracts. For Western blot analysis, monoclonal antibodies against p25 (VPM70, kindly provided by Dr B. Blacklaws, University of Cambridge, UK) was used undiluted. Anti-mouse IgG peroxidase-conjugated (Pierce) was used as secondary antibody. In addition, sera from two experimentally infected sheep with SRLV strains from genotype A and B respectively were used to reveal protein production. Reactions were developed by chemiluminescence (Amersham ECL Western blotting detection reagents; GE Healthcare, Buckinghamshire, UK).

### Neutralizing antibody (NtAb) assay

Heat inactivated sera from ELISA positive red deer (*n* = 6) were serially diluted in DMEM 2% FBS in a 96-well plate and incubated 1:1 (vol/vol) with 100 TCID_50_ of strain Ev1. Six wells were used per serum dilution. After mixing and incubating overnight at 4 °C, the mixture was added to sheep fibroblasts. After 2 h at 37 °C, 5% CO_2_, the supernatant containing the virus and serum mixture was removed and after washing DMEM 2% FBS was added. Following a 7 day incubation at 37 °C and 5% CO_2_ cytopathic effect (CPE, syncitia formation) was screened by microscopy. The NtAb titer was defined as the reciprocal of the serum dilution in which 50% of the culture wells showed no signs of infection.

## Results

### ELISA

Using the home-made ELISA based on synthetic peptides and protein G as conjugate, SRLV antibodies were detected in 14 out of 193 (7%) red deer and one mouflon out of 10 analyzed. Using a secondary antibody able to react against wild ruminant species (EG5, Ingenasa), increased the number of seropositive red deer to 20 out of 141 (14%) and, moreover, allowed the detection of SRLV antibodies in 3 out of 36 roe deer (8%) and one mouflon, but not in fallow deer. Combining results using protein G and EG5 conjugates SRLV antibodies were detected in 26 out of 259 (10%) red deer, 3 out of 36 roe deer and one out of 16 analyzed mouflon.

**Figure 2:**
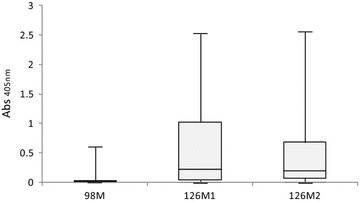
**Peptide ELISA.** Absorbance distribution (Abs_405nm_) of seropositive red deer (*n* = 11) obtained with individual peptides 98 M, 126M2 and 126M1 derived from Env protein of SRLV.

Single peptide ELISA analyses indicated that peptides 126M1 and 126M2 were responsible for the seropositivity in most of the animals (Figure 2).

In order to confirm specificity of these antibody reactions, some sera were also tested for anti-SRLV antibodies using commercial ELISA (Chekit and Elitest). None of the animals that were seropositive to the peptide ELISA were seropositive to the Elitest with protein G or commercial secondary antibody conjugates. In contrast, 2 red deer that were seronegative to the peptide ELISA were seropositive with the modified Chekit test using EG5 conjugate.

Seropositive deer were mainly found (15 out of 20 seropositive deer) in commercial farms which represents the largest sample source, followed by hunting campaigns with 4 seropositive animals. Seropositive roe deer were from hunts and the seropositive mouflon was from a zoo.

Seropositive animals were tested for the presence of neutralizing antibodies obtaining titres ranging from 3 to 40.

### Screening PCRs

Some bands of the expected size were obtained following PCR amplification using different sets of primers (Table 1). However, after purification, cloning and sequencing none corresponded to known SRLV proviral sequences, instead they matched bovine genomic sequences. Therefore, PCRs for the bovine immunodeficiency virus (BIV) were performed in DNA samples from seropositive and seronegative deer, but none of the amplified products presented a lentiviral-type sequence, they were all identified as genomic DNA.

### In vitro infection and entry assay

After being unable to demonstrate virus presence in wild ruminants by PCR in vivo, we decided to shift to in vitro experimental infection and transduction experiments. For this, as indicated in the particular experiments, DSF, FSF and BDM cultures were obtained and infected with SRLV strains belonging to genotype A (Ev1 and Swiss A4) or genotype B (CAEV-Co and Ov496). After 16 h, CPE was evident in cells from both species (red deer and fallow deer) only in the case of Ev1 virus. Since this effect may correspond to a virus over load, viral stocks were titrated again and infection was repeated with similar results.

**Figure 3:**
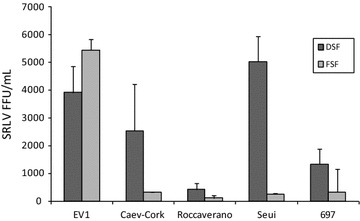
**Entry assay with pseudotyped SRLV.** Infectivity of CAEV-AP virions pseudotyped with Env proteins from Ev1, CAEV-Cork, Roccaverano, Seui and 697 SRLV strains on red deer skin fibroblasts (DSF) and fallow deer skin fibroblasts (FSF). Results are expressed as focus-forming units per milliliter (FFU/mL).

To clarify if these results correspond to a differential ability of SRLV in entering the cell, entry assays using CAEV-AP virions pseudotyped with envelopes from different SRLV strains were carried out. Although all the tested strains were able to enter into red deer and fallow deer cells, there were differences among them. Red deer cells were more susceptible to Seui, Ev1 and CAEV-Co strains in that order, while FSF mainly allowed the entrance of Ev1 strain from genotype A. Strains Roccaverano, and to a lesser extend 697, showed the lowest values in both cell types (Figure 3).

### Post entry restriction

**Figure 4:**
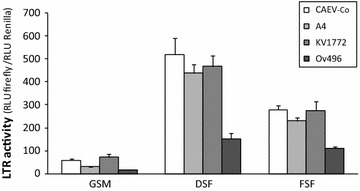
**LTR promoter activity.** LTR-U3-R region of SRLV strains CAEV-Co, A4, Kv1772 and Ov496 transfected in goat synovial membrane cells (GSM), red deer skin fibroblasts (DSF) and fallow deer skin fibroblasts (FSF). Results are expressed as the ratio between the relative firefly luminescence (RLU) and the Renilla luminescence.

Once demonstrated the SRLV ability to enter into red deer and fallow deer cells, we checked further steps of the viral cycle such as integration and RNA production. PCR at 16 h post-inoculation showed positive results in all cases confirming, the permissive entry and the presence of viral DNA within red deer and fallow deer cells. Regardless of the high transcriptional activity of the LTR in fibroblastic cells from deer and fallow deer (Figure 4), RNA production was confirmed only in the case of Ev1 infection in red deer cells 48 h after inoculation.

**Figure 5:**
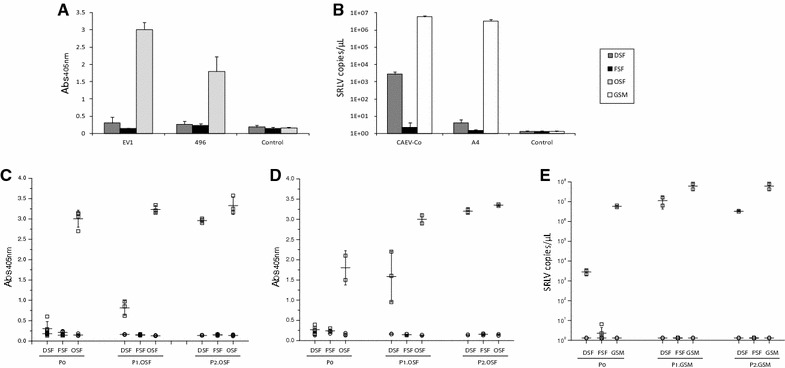
**SRLV in vitro infection upon supernatant transfer. A** RT activity (Abs_405nm_) of culture supernatants from red deer skin fibroblasts (DSF), fallow deer skin fibroblasts (FSF) and ovine fibroblasts (OSF) infected with SRLV strains Ev1 or 496. **B** Viral quantification of viral RNA (copies/µL) in the supernatants from DSF, FSF and goat synovial membrane cells (GSM) infected with strains A4 or CAEV-Co. **C, D** Culture supernatants from DSF, FSF and OSF infected with Ev1 or 496 respectively (P0) were transferred to fresh OSF twice (P1.OSF and P2.OSF) and RT values represented (Abs_405nm_). **E** SRLV RNA copies in the supernatant of DSF, FSF and GSM infected with CAEV-Co (P0) and after transfer of supernatants to fresh GSM twice (P1.GSM and P2.GSM). Squares represent values from infected cells and circles indicate non-infected cells.

In spite of these positive correlates of lentiviral productive infection, RT activity in the supernatants was negative in all cases at 7 and 10 days, suggesting low or no production of viral particles to the supernatants (Figures 5A and B). In order to check whether Ev1 and 496 viruses were completely absent from DSF and FSF supernatants, we transferred them to fresh ovine fibroblasts. After 7 days, RT activity from both viruses started to increase only in the case of DSF, reaching positive control values at the second supernatant transfer in OSF (Figures 5C and D).

In the case of strains A4 and CAEV-Co, viral RNA was detectable in the supernatants 7 days post-inoculation only in CAEV-Co infected cells. As above, supernatants from these infected-cells were used to infect GSM cells. After 7 days, viral RNA increased in the supernatant for CAEV-Co strain but remained undetectable for A4 strain (Figure 5E).

**Figure 6:**
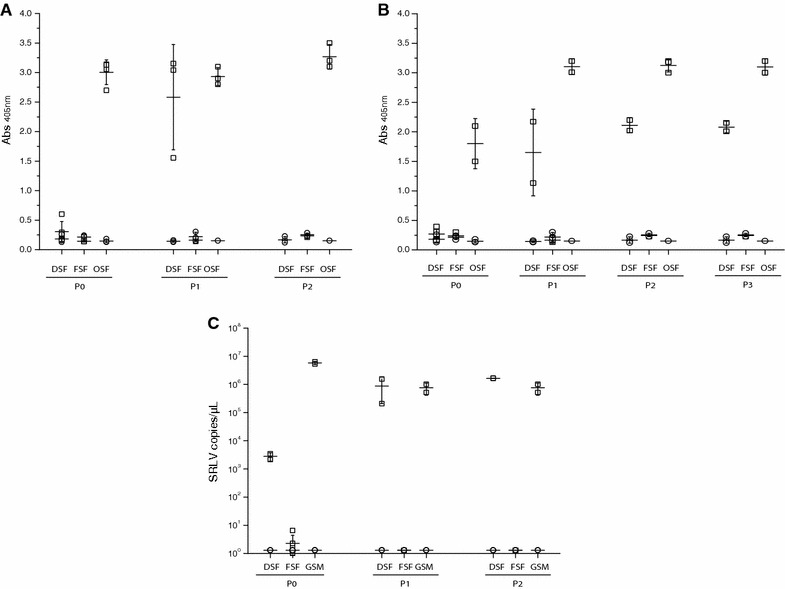
**SRLV in vitro infection upon culture passages.** RT activity in the supernatant from red deer (DSF), fallow deer (FSF) and ovine fibroblasts (OSF) infected with strains Ev1 (**A**) or 496 (**B**). **C** Viral quantification of total viral RNA (copies/µL) in the supernatants from DSF, FSF and goat synovial membrane (GSM) cells infected with strain CAEV-Co. Squares represent values from infected cells and circles indicate non-infected cells. Cell passage number is indicated (P0, P1, P2 and P3).

Furthermore, infected DSF and FSF were passed weekly by trypsinization and after the first passage strains Ev1, 496 and CAEV-Co showed increased production only in DSF, being the strain A4 not able to replicate. At the second passage, Ev1 was able to destroy the cellular monolayer whereas strain 496 was adapted to DSF causing persistent infection similar to ovine fibroblasts. Virus production after passages in FSF remained always negative (Figure 6).

**Figure 7:**
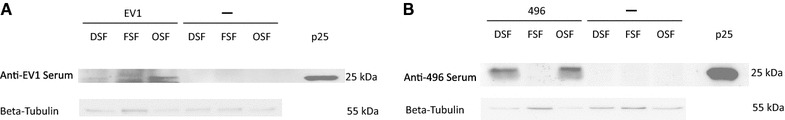
**Production of P25 protein in cell culture.** Western Blot of ovine fibroblasts (OSF) infected with supernatants from red deer (DSF), fallow deer (FSF) and OSF infected with strains Ev1 (**A**) or 496 (**B**) after 7 days of infection. Uninfected cells and recombinant p25 protein were also included as negative and positive controls respectively. Sera from two experimentally infected sheep with SRLV strains from genotype A and B respectively, were used to reveal protein production. Beta tubulin antibody was used as loading control.

Production of GAG and ENV viral proteins was evaluated by western-blot using monoclonal antibodies anti-p25 and polyclonal sera from infected sheep, aiming at improving cross-reactivity. In agreement with RT activity, capsid protein production was detected in red deer cells infected with strains Ev1 and 496 at the second passage, and in OSF after inoculation with culture supernatant from infected DSF. Also in agreement was the absence of capsid protein in FSF cells neither with 496 nor Ev1 SRLV strains (Figure 7). Env protein was not specifically detected with the antisera used.

Red deer BDM allowed Swiss A4 infection but few viral RNA copies were amplified from the supernatant. Instead, CAEV-Co replicated more efficiently reaching values obtained with ovine BDM. Fallow deer BDMs showed a more restricted phenotype, similar to that observed in skin fibroblasts showing undetectable virus production. Sheep BDM differentially allowed the replication of SRLV A4 and CAEV-Co strains. While A4 replicated at high titres, CAEV-Co RNA was produced at low levels in the supernatant, confirming the impaired replication of this virus in ovine cells (data not shown).

## Discussion

The emergence of the devastating HIV epidemics in the human population following cross species transmission from the natural non-human primate host, in which these lentiviruses cause non-pathogenic persistent infections [48], highlights the ability of this group of viruses to cross the species barrier, adapt to a new host and dramatically increase their virulence.

Transgression of the species-specific barrier by SRLV has been explored in cattle through experimental infection of cows with CAEV-Co strain [49]. SRLV infection induced a persistent antibody production, similar to that observed in sheep or goats, suggesting viral protein production. However, this was not accompanied by productive long-term infection; indeed although authors demonstrated proviral integration into leukocytes and tissues, infection did not persist more than 4 months, and virus was not recovered even after attempts to reactivate viral replication. Virus clearance was unlikely due to the humoral response, since antibodies were not neutralizing.

Here, we show positive serological reaction against different SRLV antigen preparations in red deer, roe deer and mouflon, being fallow deer negative. However, this serological reaction was not accompanied by the detection of SRLV sequences by PCR in vivo. In vitro, skin cell cultures from red deer and fallow deer did not produce detectable levels of SRLV Gag or RT proteins in spite of the presence of syncytia and viral DNA and RNA. Infection was rescued by transferring supernatants from red deer skin fibroblasts infected in vitro, to fully permissive ovine fibroblasts suggesting the production of low levels of virus. Although highly dependent on the infecting strain, virus adaptation could be induced in DSF through periodical cell passages in vitro. In contrast, fallow deer cells did not support SRLV infection despite supernatant transfer or cell passages in agreement with serological findings. These results may suggest that SRLV are at different steps in the viral adaptation to red deer and fallow deer. In the last case, host may have evolved to mount an effective long-lasting restriction mechanism of lentiviral replication in vivo. Neutralizing antibodies were detected in ELISA positive sera from red deer however, they unlikely play an important role in the viral infection restrictive pathway as described in caprine and ovine species [21, 50]. Serological reaction was mostly detected by home-made ELISA in comparison with commercial tests as described for ovine and caprine infections likely due to the inclusion of novel epitopes providing wider cross-reactivity [39, 51]. Reaction was mainly directed against peptides 126M1 and 126M2 suggesting a potential SRLV infection involving multiple genotypes. Synthetic peptide ELISA performance was better using secondary antibody than protein G whose specificity against IgG from sheep and goats is moderate and unknown in red deer [52]. Contact with sheep or goats cannot be excluded in animals coming from hunts or national parks, however seropositive animals were mainly found in commercial farms and zoos in which these contacts are at present highly restricted.

Intra-individual SRLV viral reservoir is represented by proviral integration into monocytes that after maturation to tissue macrophages become permissive to viral replication. Wild ruminants could act as inter-individual reservoir potentially responsible for the re-emergence of infection in domestic small ruminant flocks [32]. The high SRLV genome plasticity is translated into a wide tropism that allows the generation of immune escape mutants and the colonization of new target cells and hosts [53]. Accordingly, cells from humans, monkey, hamster, mice, quail, cows [49, 54, 55] and, as shown in this study, red deer and fallow deer are permissive to SRLV entry since viral DNA was detected in vitro upon infection of DSF and FSF. Consequently, amplification and adaptation to a broad spectrum of host species could be expected. Indeed, SRLV are causing natural cross-species infection in wild ruminants following contact during the free grassing season in wilderness areas, potentially generating uncontrollable reservoir of viruses [32]. This could eventually represent a major obstacle in SRLV eradication programs in domestic sheep and goat flocks.

Endogenous retroviruses from cervids (CERVs) have been described in Mule deer (*Odocoileus hemionus*) genome [56], confirming previous contacts with retroviruses. Strong humoral and T cell responses are elicited against human retroviruses, especially to HERV-K10 in healthy but mostly in cancer patients due to higher viral expression [57, 58]. However, small ruminants do not mount a strong humoral or cellular immune response against exogenous JSRV, likely due to the presence of a related endogenous counterpart that induce tolerance [59]. Interestingly, sequences from CERV *gag* region were compared with VMV-like isolates and preliminary analyses indicate some degree of structural similarity between both sets of sequences that might explain the presence of serological reaction in the absence of an exogenous lentivirus in vivo. Unfortunately, it is unknown whether red deer develop a detectable antibody production against CERV.

Antigenic cross-reactivity with CAEV in humans has been reported likely due to the consumption of contaminated caprine dairy products [60] potentially favoring SRLV adaptation to humans. In fact, numerous emerging infectious diseases including zoonosis have been originated from wildlife [1, 61]. Domestic animals have been selected for centuries towards a specific production (milk, meat, wool, etc.) and have not been subjected to the same natural selection pressure as their wildlife counterparts and as a result, they are less resistant to a high number of pathogens [62]. However, repeated contacts of potentially susceptible wildlife with domestic SRLVs may lead to the emergence of new adapted lentivirus variants [32].

Lack of viral proteins in cultured cells from wild ruminants could be a consequence of the abnormal processing of the Env protein in these cell types. This has been previously described in sheep choroid plexus cells infected with CAEV-like virus [63]. However, if this was the case no CPE would be expected in DSF cells. Moreover, in vitro findings in this study clearly show the presence of newly synthesized infectious particles in DSF and the ability of SRLV to adapt rapidly to cells from wild ruminants in vitro. However, since in vivo lentivirus cross-species transmission is a rare event that only occurs under specific circumstances other factors related to the host immune response may hamper the progression of infection.

The host innate immunity may play an important role in counteracting infection independent of the presence of antibodies which are generated following antigenic presentation in the presence or absence of viral replication. Remarkably, cell factors involved in innate and adaptive immunity may evolve much quicker than those required for cell survival likely contributing to lentiviral resistance. Intracellular restriction mechanisms described in small ruminants include tripartite motif containing (TRIM5α) and APOBEC3, both leading to proteasomal degradation of viral proteins and therefore sharing the MHC-I antigenic presentation pathway that could result in antibody production in the absence of viral replication. On the other hand, tetherin, which impedes normal virus burden at final steps of the infection cycle, may interfere virus release without affecting proviral load or RNA transcripts. Therefore a future study on SRLV restriction due to intrinsic factors from wild ruminants is warranted.

In summary, our results suggest that cells from the Cervidae family are susceptible to SRLV infection in vitro but factors involved at post-entry steps are controlling infection spread in vivo. These results may indicate that cervids have a bystander role in SRLV epidemiology.
